# Profil épidémio-clinique des dermatoses chez les enfants vus en consultation dermatologique dans le service de dermatologie du centre national d’appui à la lutte contre la maladie à Bamako (Mali)

**DOI:** 10.11604/pamj.2016.25.238.10564

**Published:** 2016-12-20

**Authors:** Youssouf Fofana, Bekaye Traore, Adama Dicko, Ousmane Faye, Siritio Berthe, Lamissa Cisse, Alimata Keita, Koureissi Tall, Mamadou Bakary Kone, Somita Keita

**Affiliations:** 1 Centre National d’appui à la Lutte contre la Maladie à Bamako, Mali

**Keywords:** Dermatoses, infantiles, dermato-vénérologie, Pediatric dermatoses, dermatology, venereology

## Abstract

**Introduction:**

Les maladies de peau constituent un problème majeur de santé publique dans les pays en voie développement. En pratique courante les enfants représentent la couche sociale la plus touchée. Le but de notre travail était de décrire les aspects épidémio- cliniques des dermatoses chez les enfants de 0-15 ans dans le service de dermatologie du centre national d’appui à la lutte contre la maladie à Bamako, Mali.

**Méthodes:**

Il s’agissait d’une étude transversale effectuée au cours de la période allant du premier janvier 2009 au 31 Décembre 2009 dans le service de Dermato-vénérologie du centre national d’appui à la lutte contre la maladie. Sur un total de 16339 patients ayant fait une consultation dermatologique 5149 enfants ont été inclus.

**Résultats:**

La fréquence hospitalière des dermatoses infantiles était de 31,51%. Les malades se répartissaient en 2838 garçons (55,10%) et 2311 filles (44,90%) soit un sex-ratio de 1,22. L’âge des malades variait de 03 jours à 15 ans avec une moyenne d’âge de 8±5,7 ans. Parmi les affections retrouvées les dermatoses infectieuses représentaient 55,10% de l’ensemble des dermatoses, les dermatoses immuno-allergiques (32,5%), les dermatoses inflammatoires (11,85%).

**Conclusion:**

Notre étude a révélé l’importance des pathologies infectieuses et immuno-allergiques et la nécessité de mener des actions de prévention simple comme l’hygiène, l’achat d’une tondeuse pour chaque enfant.

## Abstract (en)

**Introduction:**

Skin diseases are a major public health problem in developing countries. In common practice, children represent the most affected segment of the population. This study aims to describe the epidemiological and clinical aspects of skin diseases in children aged 0-15 years receiving dermatological consultation in the Department of Dermatology at the National Center for Disease Control in Bamako (Mali).

**Methods:**

We conducted a cross-sectional study in the Department of Dermatology and Venereology at the National Center for Disease Control from 1 January 2009 to 31 December 2009.Out of a total of 16339 patients who had undergone a dermatological consultation 5149 children were included in the study.

**Results:**

Dermatitis-frequency in hospital attending children was 31.51%. We enrolled 2838 boys (55,10%) and 2311 girls (44.90%), with a sex-ratio of 1,22. Patient age ranged from 03 days to 15 years with an average age of 8 ± 5.7 years. Infectious dermatoses accounted for 55.10% of all dermatoses, immuno-allergic dermatoses (32.5%), inflammatory dermatoses (11.85%).

**Conclusion:**

Our study highlights the severity of infectious and immuno-allergic pathologies and the necessity to promote simple preventive measures such as hygiene, buying a clipper for each child.

## Introduction

Les maladies de peau constituent un problème de santé dans les pays en voie développement [1, 2]. Dans la population générale les enfants représentent la couche sociale la plus touchée. Dans les centres de santé, les maladies de peau représentent le 3e motif de consultation après les fièvres présumées palustres et les maladies diarrhéiques [3]. Ainsi les complications liées à ces dermatoses sont multiples et peuvent être très sévères mettant parfois en jeu le pronostic vital. La glomérulonéphrite aigue (GNA) représente une complication fréquente de la pyodermite, elle peut conduire à une insuffisance rénale voire au décès. A cela, il faut ajouter le fort pourcentage d’infections cutanées dans l’étiologie des fièvres aigues bactériennes chez l’enfant. Paradoxalement, l’étude des registres de consultation de ces agents montre des indices concordant sur la mauvaise qualité de la prise en charge de ces affections. Très peu d’études ont été réalisées sur les dermatoses pédiatriques au Mali, raison pour laquelle ce travail a été initié. Le but de notre travail était de décrire les aspects épidémio-cliniques des dermatoses chez les enfants de moins de 16 ans en milieu hospitalier dermatologique à Bamako.

## Méthodes

Du 1er Janvier 2009 au 31 Décembre 2009, nous avons mené une étude transversale des cas de dermatoses infantiles. L’étude a été réalisée dans le service de dermatologie du Centre National d’Appui à la lutte contre la Maladie (CNAM-ex institut Marchoux). Ce service situé dans la capitale du Mali (Bamako) représente le seul centre de référence dermatologique du pays. L’enquête a consisté à recenser, au cours de la consultation, tous les enfants présentant une dermatose. Ainsi, ont été inclus tous les enfants quelque soit l’âge et le sexe. Les enfants étaient examinés par un dermatologue en présence d’un accompagnant, qui répondait aux questions posées par l’enquêteur.

Le diagnostic dermatologique était basé sur l’examen clinique complété au besoin par des examens biologiques ou l’histopathologie cutanée. Les données ont été recueillies sur un questionnaire à partir des dossiers médicaux qui comportaient les variables démographiques (âge, sexe, scolarité), cliniques (motif de consultation, diagnostic clinique, complications) et biologiques (tests sanguins, histopathologie cutanée, examens bactériologiques et mycologiques). Le logiciel SPSS 12.0 a été utilisé pour la saisie et l’analyse des données.

## Résultats

Durant la période d’étude, 5149 enfants de moins de 16 ans ont été inclus sur un total de 16339 consultants soit une fréquence de 31,51%. Les malades se répartissaient en 2838 garçons (55,10%) et 2311 filles (44,90%) soit un sex-ratio de 1,22. L’âge des malades variait de 03 jours à 15 ans avec une moyenne d’âge de 8±5,7 ans; les enfants d’âge préscolaire (moins de 5 ans) représentait 58,3% (3003/5149), Les enfants scolarisés représentaient 30,12% soit 1551 sur 5149 (Tableau 1). Sur le plan clinique, le prurit était le principal motif de consultation chez 89,75% des malades (3886/5149) (Tableau 2). Les principales affections recensées étaient les dermatoses infectieuses (55,10%), les dermatoses immuno-allergiques (32,50%), les dermatoses inflammatoires (11,85%), les génodermatoses (0,50%), les maladies dysimmunitaires (0,01%).

**Tableau 1:** Répartition des patients selon le groupe d’âge

Tranche d’âge	Effectifs	Pourcentage
0-5 ans	3003	58,3
6-10 ans	1261	24,5
11-15 ans	885	17,2
Total	5149	100

Parmi les dermatoses infectieuses, nous avions noté des mycoses qui représentaient 31,68% (1631/5149) constituées par la teigne 17,3% (892/5149), la dermatophytie 6,9% (357/5149) et la dermite séborrhéique (4,4%). En ce qui concerne les dermatoses bactériennes, elles étaient présentes chez 12,74% des malades (656/514), comprenaient l’impétigo (12,2%), l’érysipèle (0,4%) et la lèpre (0,1%) (Tableau 3). Les infections virales constituaient 8,4% des pathologies (434/5149), et comprenaient le molluscum contagiosum (4%), le pityriasis Roger de Gibert (3,1%), les verrues planes (0,8%) (Tableau 3). Quant aux ectoparasitoses, elles incluaient 60 cas de larva migrans (2,3%) et 57 cas de scabiose (1,2%) (Tableau 3).

**Tableau 2:** Répartition des patients selon les motifs de consultation

Signes fonctionnels	Effectifs	pourcentage
Prurit	4621	89,75
Douleur	444	8,62
Fièvre	84	1,63
Total	5149	100

**Tableau 3:** Répartition des patients selon les dermatoses infectieuses

Dermatoses infectieuses	Pathologies	Effectifs	pourcentage
Mycoses	Teigne	892	17,3%
	Dermatophytie peau glabre	357	6,9%
	Dermite séborrhéique	226	4,4%
	Candidoses	123	2,4%
	Pityriasis versicolor	33	0,6%
	Total	1631	31,68%
Bactériennes	Impétigo	626	12,2%
	Erysipèle	23	0,4%
	Lèpre	7	0,1%
	Total	656	12,74%
Virales	Molluscum contagiosum	208	4%
	PRG	158	3,1%
	Verrues planes	40	0,8%
	Varicelle	17	0,3%
	Zona	11	0,2%
	Total	434	8,4%
Ectoparasitoses	Larvan migrans	60	1,2%
	Scabiose	57	1,1%
	Total	117	2,3%
Total		2838	55,10%

Les dermatoses immunoallergiques representaient 32,7% de l’ensemble des dermatoses et étaient dominées par la dermatite atopique (15,6%), le prurigo (9,50%), l’eczéma de contact (3,50%), le vitiligo (2,9%) (Tableau 4). Les dermatoses inflammatoires avaient été évoquées chez 11,85% des malades (610/5149). Elles incluaient 386 cas de kératodermies palmo-plantaires (7,49%), 105 cas de dermite de couche (2%), 41 cas d’acné (0,8%), 16 cas de psoriasis (0,3%) et 25 cas de lichen (0,5%).

**Tableau 4:** Répartition des patients selon les dermatoses immunoallergiques

Dermatoses immunoallergiques	Effectifs	Pourcentage
Eczéma atopique	803	15,6
Prurigo	487	9,5
Eczéma de contact	178	3,5
Vitiligo	148	2,9
Urticaire	34	0,7
Toxidermie	13	0,3
PRP	9	0,2
Total	1672	32,5

Dans la catégorie des génodermatoses, on notait 7 cas d’ichtyose congénitale (0,1%), 7 cas de naevus (0,1%) et 3 cas neurofibromatose (0,05%). Parmi les maladies dysimmunitaires 1 seul cas de lupus a été retrouvé. Les localisations de ces affections étaient variées et concernaient la tête chez 36,88% des malades (3398/5149), les membres chez 33,24% des patients (3062/5149), le tronc chez 12,34% des patients (1137/5149), le cou chez des patients 3,79% (349/5149), les régions génitales chez 2,25% des patients (207/5149), les fesses chez des patients 5,20% (478/5149) et les plis chez 6,30% des patients (581/5149).

## Discussion

Le but de ce travail était de décrire les aspects épidémiologique et clinique des dermatoses chez les enfants de moins de 16 ans vus en consultation dermatologique à Bamako. L’étude a été réalisée dans le plus grand centre de référence dermatologique du pays et sur la cohorte de consultants d’une année. Les diagnostics retenus étaient essentiellement basés sur l’examen clinique, l’on a eu recours, dans un petit nombre de cas, à des examens biologiques (histopathologie cutanée, tests sanguins, examens mycologique et bactériologique).

Notre travail apporte une contribution significative à l’épidémiologie et l’étude clinique des dermatoses courantes chez les enfants. Ainsi, dans notre étude, la fréquence des dermatoses infantiles était de 31,51%. Ceci va dans le sens d’une étude réalisée à Bamako par Mahé et col qui trouvaient 32,9% [4]. L’écrasante majorité des demandes de soins était formulée par les enfants âgés de 03 jours à 5 ans (58,3%), contrairement à une série turque ou les adolescents étaient majoritairement représentés (47,6%) [5]. Les garçons dans notre série ont été les plus nombreux et ce résultat va dans le sens d’une étude réalisée à Conakry en 2011 qui rapportait un sex-ratio de 1,21 M/F [6].

Cette fréquence élevée pourrait être la conséquence d’une hygiène défectueuse, des conditions socioéconomiques médiocres et l’insuffisance des soins médicaux [7]. Sur le plan clinique, le prurit était le principal motif de consultation chez les enfants dans notre étude (89,75%), cela s’explique par le nombre important des dermatoses prurigineuses à savoir : mycoses, l’eczéma atopique et l’impétigo. Des résultats similaires ont été apportés dans la littérature [6]. Dans notre étude, les dermatoses mycosiques ont représenté 31,68% de l’ensemble des dermatoses infectieuses. Ces résultats vont dans le sens des travaux antérieurs [3, 6]. En ce qui concerne les teignes, elles représentaient (17,3%), supérieurs au résultat trouvé par Mahé et col qui était de 5,3% [4]. Donc l’usage de la tondeuse unique à l’occasion des fêtes pour coiffer les enfants est le principal facteur de contamination. Les dermatoses bactériennes étaient dominées par l’impétigo (12,2%). Cela ne rejoint pas les résultats de la série guinéenne ou les folliculites étaient les plus fréquentes (60,27%) [6]. Elles sont surtout favorisées par le manque d’hygiène, la difficulté de l’accès à l’eau potable, la promiscuité et la malnutrition. En ce qui concerne les dermatoses immuno-allergiques, l’eczéma atopique et le prurigo étaient les pathologies dominantes. Ces résultats vont dans le sens d’une étude réalisé au bénin ou les dermatoses immuno-allergiques représentaient 37% des motifs de consultation [8]. L’urbanisation galopante, la promiscuité et les habitats confinés participent à la prévalence élevée de ces affections immuno-allergiques.

Les dermatoses inflammatoires étaient dominées par les kératodermies palmo-plantaires. Ceci contraste avec le résultat trouvé par Mahé et col qui était de 3,6% [3]. Cela s’explique par l’errance et la marche sans port de chaussure. La région céphalique et les membres ont été les sièges de prédilection des différentes dermatoses. Cette observation avait été constatée par certains auteurs comme D. Ye et al au Burkina faso [9]. Cela peut s’expliquer par leur exposition aux agressions externes, physiques, climatiques etc.

## Conclusion

Les maladies de peau sont fréquentes dans la population pédiatrique. Notre étude a révélé l’importance des affections dermatologiques en particulier infectieuses et immuno-allergiques et la nécessité de mener des actions de prévention simple comme l’hygiène, l’achat d’une tondeuse pour chaque enfant.

### Etat des connaissances actuelles sur le sujet

- Véritable préoccupation pour les médecins;
- Les enfants peuvent présenter une grande variété de dermatoses.

### Contribution de notre étude à la connaissance

- Connaissance de la fréquence des dermatoses infantiles au Mali;
- Les différentes dermatoses infantiles au Mali.
